# Equity or Two-Tier Care? Guardrails for Silver Diamine Fluoride and Delegated Early Childhood Caries Pathways

**DOI:** 10.3390/children13030386

**Published:** 2026-03-10

**Authors:** Ziad D. Baghdadi

**Affiliations:** 1 Centre for Community Oral Health, Dr. Gerald Niznick College of Dentistry, University of Manitoba, Winnipeg, MB R3B 0L8, Canada; drziadeddin.albaghdadi@gmail.com or zalbaghdadi@atsu.edu; 2 Department of Preventive Dental Sciences, Division of Pediatric Dentistry, Dr. Gerald Niznick College of Dentistry, University of Manitoba, Winnipeg, MB R3E 0W2, Canada; 3 TopSmiles Pediatric Dentistry and Orthodontics, Winnipeg, MB R2M 3A4, Canada; 4 Butterfly Dental Group, Winnipeg, MB R3Y 1P5, Canada

**Keywords:** early childhood caries, silver diamine fluoride, pediatric dentistry, delegated care, caries risk assessment, implementation science, health equity, evidence translation, oral health–related quality of life, dental home

## Abstract

Early childhood caries (ECC) is a complex, multifactorial disease shaped by biofilm ecology, host susceptibility, diet and behaviors, and structural determinants of health. Silver diamine fluoride (SDF) is an effective non-restorative option for arresting cavitated lesions in many settings and can support access when definitive care is delayed. However, translating short-horizon “arrest” outcomes into broad policy claims—that SDF-first, delegated pathways can substitute for dentist-led diagnosis and comprehensive rehabilitation—risks institutionalizing a two-tier standard of care for children facing the greatest access barriers. This perspective critically appraises evidence-to-implementation pathways for SDF and delegated ECC management, using risk-of-bias and reporting guidance as interpretive tools and drawing on pragmatic regimen trials, microbiome substudies, oral health-related quality of life (OHRQoL) analyses, and implementation work including the Canadian Caries Risk Assessment Tool (CCRAT) in primary care. We explicitly distinguish what studies demonstrate (e.g., feasibility and short-term arrest differences by reapplication interval) from what they do not establish (e.g., long-term tooth survival, pulpal outcomes, definitive treatment completion, and equity impacts). We propose practical guardrails that position SDF as **interim management within a continuum of care**: dentist-led diagnosis and escalation when pulpal risk is suspected; time-bound referral pathways with completion tracking; protocolized follow-up aligned with lesion/risk status; outcome sets that extend beyond “arrest” to include pain, function, OHRQoL, tooth survival, and equity stratification; and lesion-site sampling plus preregistered analyses when mechanistic claims are advanced.

## 1. Introduction: From “Complex Disease” to Simplified Pathways

Early childhood caries (ECC) is widely understood as a multifactorial disease that arises from interactions among biological, behavioral, and structural determinants. Conceptual models emphasize that children’s oral health outcomes are shaped by influences at multiple levels, including the child, the family, the community, and the health system. At the biological level, ECC reflects the ecology of dental plaque and biofilm community dynamics, which are modified by host factors and exposures, such as frequent consumption of dietary sugars, oral hygiene practices, and fluoride access. At the structural level, access barriers, poverty, rurality, and historic inequities influence both risk and the ability to complete timely treatment. These elements co-occur and amplify each other rather than acting independently [1,2,3,4].

This article is a Perspective/Commentary. Its purpose is not to conduct a systematic review. Rather, it offers a critical conceptual appraisal of how ECC evidence—particularly evidence on silver diamine fluoride (SDF) and delegated pathways for caries management—is being translated into implementation programs and policy narratives. To support transparent interpretation, we draw on established principles from risk-of-bias and reporting guidance (e.g., RoB 2, CONSORT, STROBE) as interpretive tools to help distinguish (i) what specific studies demonstrate from (ii) what is being inferred for population-scale models of care [5,6,7,8].

SDF is increasingly used for non-restorative management of cavitated lesions, often framed as “caries arrest.” Clinical guidelines and high-level syntheses recognize SDF as effective for arresting lesions in many contexts, while also emphasizing trade-offs (notably black staining), heterogeneity in evidence, and the need for follow-up and care pathways rather than single-visit “cures” [9,10,11,12,13]. When SDF is presented as an interim measure within a structured pathway, it can reduce pain risk, buy time, and support access where definitive care is delayed.

The implementation challenge arises when short-horizon study outcomes (e.g., “arrest” at the next visit) become treated as a substitute for comprehensive outcomes (e.g., tooth survival, pulpal health, function, and completed definitive care), and when systems interpret SDF-first care as a justification to lower diagnostic and treatment infrastructure for children who already face the greatest access barriers. This Perspective examines “evidence-to-implementation drift,” and proposes guardrails to prevent SDF-first and delegated approaches from becoming a de facto two-tier standard.

## 2. Why Simplified Models Spread and Why Guardrails Matter

Simplified delivery models are attractive because they appear scalable: they reduce chair time, can be delivered outside dental clinics, and can be coupled with brief screening or risk tools. In contexts where operating room dental rehabilitation is common, clinician shortages exist, and families face long wait times, SDF can function as a pragmatic bridge.

However, scale and simplicity can lead to unintended consequences when implementation logic exceeds evidentiary boundaries. Several recurrent risks deserve explicit attention:

1. **Endpoint substitution:** short-term “arrest” is treated as treatment completion, even when function, symptoms, and long-term outcomes remain unknown.
2. **Scope substitution:** screening tools and checklists are treated as a diagnosis, and delegated delivery is treated as equivalent to dentist-led treatment planning.
3. **Equity inversion:** innovations intended to improve access can inadvertently normalize reduced care options for populations with the greatest burden, while children with proximity and privilege still receive comprehensive rehabilitation.

These risks do not argue against SDF or integrated care. They argue for a disciplined translation pathway: clear case selection, structured follow-up, escalation criteria, accountability for definitive care completion, and outcomes that reflect what matters to children and families.

## 3. What the SDF Evidence Supports and What It Does Not Establish

### 3.1. What the Evidence Supports

High-level syntheses conclude that SDF is effective for arresting carious lesions under many conditions, and guidelines support SDF as a non-restorative option for managing carious lesions with appropriate counseling and follow-up [9,10,11,12,13]. Importantly, many guidelines frame SDF as part of a broader management strategy rather than as a stand-alone solution. The evidence base also suggests that frequency/regimen matters, although effects vary by population, setting, lesion type, and co-interventions, and the certainty of evidence differs across outcomes [9,10,14].

### 3.2. What the Evidence Does Not Establish (Boundaries)

Even where SDF improves short-term arrest metrics, many studies are not designed to establish:

- **Long-term tooth survival** and reactivation rates over clinically meaningful horizons;
- **Pulpal outcomes** (e.g., progression to symptoms, need for urgent care);
- **Functional rehabilitation** (e.g., restored chewing, sleep, nutrition);
- **Completion of definitive care**, where indicated (e.g., restorations, Hall technique, extraction, comprehensive rehabilitation);
- **Equity impact**, including whether SDF-first delivery reduces or increases time-to-treatment completion in high-burden populations.

This is not a critique of SDF; it is a reminder about inference boundaries. Policies that treat arrest metrics as a substitute for comprehensive outcomes require additional evidence and explicit safeguards.

### 3.3. Why This Distinction Matters for Implementation

When “arrest” becomes the operational currency of success, systems can unintentionally shift from *bridging care* to *repeating interim care*. This is especially relevant where program incentives reward volume and visible short-horizon endpoints rather than completion of treatment pathways.

## 4. Evidence Appraisal I: SDF Regimen Trials and the Risks of Over-Translation

This section uses a recent open-label randomized trial of different SDF application intervals as a worked example of how a legitimate pragmatic trial question can be over-extended in implementation narratives [14].

### 4.1. What the Regimen Trial Directly Demonstrates

In a Canadian open-label, parallel-group randomized clinical trial, preschool children with cavitated lesions received 38% SDF plus 5% sodium fluoride (NaF) varnish with varying reapplication intervals. The study reports higher short-term arrest in shorter-interval arms at the end of follow-up [14].

This supports a bounded inference: the reapplication interval can influence short-term arrest under the trial conditions.

### 4.2. What the Regimen Trial Does Not Establish

The design and horizon do not establish a short-interval SDF regimen:

- Replaces the need for dentist-led diagnosis and treatment planning;
- Prevents pulpal progression over longer horizons;
- Reduces the need for operating room rehabilitation;
- Achieves durable outcomes without structured escalation and definitive care completion;
- Improves equity in treatment completion.

This boundary is important because regimen findings are sometimes interpreted as an implementation mandate rather than as context-dependent operational data.

### 4.3. Interpretation Considerations (Risk-of-Bias and Reporting Principles as Guardrails)

Open-label pragmatic trials are valuable, but they require careful interpretation when endpoints are subjective or carry a treatment signature.

- **Outcome assessment vulnerability:** “Arrest” is often judged by tactile hardness and color. Because black staining is an expected effect of SDF, unblinded assessment can introduce incorporation bias when “black and hard” is treated as confirmation of arrest [5,6,7].
- **Clustering and unit of analysis:** ECC trials often include multiple lesions within the same child; lesion-level analyses require multilevel modeling to avoid overstating precision.
- **Short-horizon limitations:** Short follow-up captures early changes, but not longer-term survival, reactivation, pulpal outcomes, and downstream treatment completion.
  These issues do not invalidate the trial; they clarify what claims it can support. Short-horizon limitations must also be considered when interpreting regimen trials. Large trials in other settings have also examined SDF regimens and comparisons in preschool populations [15,16].

### 4.4. International Context: Frequency Effects Are Not New, and Acceptability Matters

Large trials in other settings have examined SDF regimens and comparisons, including kindergarten-based contexts, showing that regimen effects exist but are intertwined with staining, acceptability, oral health-related quality of life, and setting-specific realities [13,15,16,17,18]. High-level syntheses continue to emphasize heterogeneity across outcomes and regimens, supporting the position that frequency results should not automatically translate into universal protocols without pathway guardrails [9,10].

### 4.5. Implementation Implication (Bounded)

A practical interpretation consistent with evidence boundaries is:

- Use regimen trial results to inform follow-up planning and resource design;
- Avoid treating regimen differences as justification for SDF-only care or for substituting SDF for dentist-led diagnosis and comprehensive pathways.

## 5. Evidence Appraisal II: Microbiome Substudies and Mechanistic Claims

Mechanistic language can strengthen (or overstate) implementation narratives. Microbiome substudies linked to SDF regimen work are, therefore, important to interpret with clear sampling and inference boundaries.

### 5.1. What the Microbiome Trial Demonstrates

Consistent with microbiome reporting guidance [19] and earlier pilot work on microbiome change after SDF [20], a randomized clinical trial evaluated changes in the bacteriome and mycobiome associated with SDF regimens, sampling supragingival plaque over time [21]. The work contributes hypothesis-generating observations about overall plaque ecology under combined preventive regimens [20,21].

### 5.2. What It Does Not Establish (And Why Sampling Frames Matter)

The sampling approach was not lesion-site specific, limiting lesion-level inference. Transparent reporting standards for microbiome research emphasize clarity regarding sampling strategy, processing, and analytic reproducibility [19]. Prior microbiome work following SDF treatment illustrates that community-level patterns can shift even when broad diversity metrics appear relatively stable [20]. When studies sample broadly across tooth surfaces but interpret shifts as if they represent lesion-level transformation, the inference can exceed the sampling frame. Transparent reporting standards for microbiome research emphasize clarity regarding sampling strategy, processing, and analytic reproducibility—particularly when mechanistic claims could influence clinical scope decisions [19].

Aligning sampling with inference is therefore essential when mechanistic claims are advanced [19,20].

### 5.3. Implementation Implication (Bounded)

Microbiome findings should be framed as exploratory ecological evidence unless lesion-site sampling and analysis justify lesion-level mechanistic conclusions. Mechanistic language should not be used as a substitute for clinical outcome evidence or as a justification to simplify care pathways.

## 6. The Delegation Axis: Screening and Risk Tools in Primary Care

Delegated approaches frequently rely on screening or risk tools embedded in medical settings to identify children at risk and trigger preventive actions.

### 6.1. The Canadian Caries Risk Assessment Tool: Promise and Limits

The Canadian Caries Risk Assessment Tool (CCRAT) was designed to support caries risk assessment and early identification, including use by non-dental primary care providers [22,23,24,25]. Integration into early-childhood frameworks can expand contact opportunities if pathways to definitive dental care exist [23,24,25,26,27] and if timely first dental visits are reinforced [28].

A pilot validation study reports high sensitivity with modest specificity for predicting new cavitated lesions [22], consistent with broader cautions about the predictive limits of caries risk concepts [29]. As with many screening-type tools, sensitivity supports early identification, but low specificity can label many children as high risk who may not progress as predicted. This is not inherently problematic; it becomes problematic when a screening tool is interpreted as a substitute for diagnosis or used to justify delegated management without an infrastructure for dental assessment and treatment completion.

### 6.2. What Observational Validation Can and Cannot Support (STROBE as an Interpretive Lens)

Observational validation and implementation studies are essential, but their interpretive limits must be explicit. Reporting guidance highlights the importance of setting, examiner characteristics, and generalizability [8].

Key boundary questions include:

- Was validation conducted in the intended setting (primary care) or in dental clinics?
- Were assessors blinded to CCRAT inputs?
- Do performance characteristics translate to populations with different baseline risk and access constraints?

These are not technicalities; they affect whether an implementation model improves care or expands administrative labeling.

### 6.3. Implementation Reality: Training Needs and Workflow Barriers

Recent evidence underscores that implementation is not simply the presence of a tool, but training, workflow, communication, and system linkage.

- Training-needs work reports that providers view tools as feasible but emphasize the need for culturally appropriate, hands-on education to implement caries risk assessment competently [26].
- Qualitative work in Indigenous pediatric primary care identifies structural barriers and a communication risk: families may interpret preventive actions as equivalent to “dental care,” potentially delaying definitive dental visits when pathways are not explicit [27].

These findings reinforce the need for delegated models to include explicit “stop points” and escalation pathways—not just screening.

## 7. Separating Evidence Appraisal from Policy Interpretation

A recurring challenge in ECC policy discourse is the unacknowledged transition from study-level findings to program-scale claims. A useful way to prevent category errors is to formalize the translation chain:

1. **Study question and endpoint** (e.g., short-horizon arrest under specific conditions);
2. **Bounded inference** (what can reasonably be generalized);
3. **Implementation claim** (what the system proposes to do at scale);
4. **Equity implication** (who receives what standard of care).

Problems arise when step 1 is treated as automatically justifying step 3. For example:

- A regimen trial showing differences in short-term arrest does not establish that SDF can replace diagnosis, pulpal assessment, or comprehensive rehabilitation [14].
- Non-site-specific microbiome studies do not establish lesion-level mechanistic transformation sufficient to justify simplified care [20,21].
- A screening tool with modest specificity does not establish safe delegation of management without adequate infrastructure and accountability for completing definitive care [22,25,26,27,29].

This Perspective argues that implementation should proceed only when the inference chain is explicit and when guardrails address the predictable failure modes.

## 8. What Responsible Implementation Looks Like: Guardrails for SDF-First and Delegated ECC Pathways

The goal is not to reject simplified approaches, but to discipline them so that access innovations expand care without lowering standards.

### 8.1. Clinical Guardrails for SDF in ECC

SDF should be positioned as interim management within a continuum of care, not as a substitute for care completion. Practical guardrails include:

- **Dentist-led diagnostic accountability** for cavitated lesions where treatment planning is required, especially when pulpal risk is possible.
- **Case selection** that explicitly excludes lesions/children with signs suggesting more advanced disease requiring urgent evaluation.
- **Protocolized follow-up**, with timing aligned to lesion severity and overall risk (rather than “apply once and discharge”).
- **Shared decision making that addresses staining**, acceptability, and alternatives [9,10,11,12,13].

*Operational examples (illustrative, adaptable to local systems):*

- Confirm response and reinforce pathway engagement within 4–8 weeks after initial SDF in high-risk children;
- Schedule reapplication and reassessment at 3–6 months depending on risk, lesion activity, and access;
- Define escalation triggers (non-arrest, recurrent symptoms, caregiver concern, functional impact) that prompt dentist assessment and definitive planning.

### 8.2. System Guardrails for Delegated Pathways (Screening/Risk Tools)

Delegation can expand contact, but should not shift diagnosis or normalize incomplete pathways. System guardrails include:

- **CCRAT as identification—not diagnosis:** tools should trigger pathway activation, not replace dentist-led planning [22,23,24,25,26,27,28,29].
- **Time-bound referral pathways with completion tracking:** success should be measured as **completed dental assessment/treatment** rather than “referrals issued.”
- **Competency-based training and cultural safety:** training should include scope boundaries and escalation criteria, not only tool completion [26,27].
- **Communication standards:** parents/caregivers should be explicitly informed that screening/varnish/SDF does not equal completed dental care when definitive care is indicated [27].

*Operational Examples (Illustrative):*

- Specify referral completion targets (e.g., “dental assessment completed within 4–12 weeks for high-risk asymptomatic children,” with faster timelines when symptomatic), and audit completion rates.

### 8.3. Outcomes Guardrails: What Must Be Measured

If policy is shaped by evidence, outcomes must reflect the goals of care:

- Beyond “arrest”: pain episodes, sleep disruption, feeding/function, unplanned visits, tooth survival, reactivation, and time-to-definitive care;
- Patient-/family-reported outcomes: oral health-related quality of life (OHRQoL) using validated instruments (e.g., ECOHIS), analyzed transparently by domains rather than only total scores [30,31,32];
- Acceptability and stigma considerations, including staining and caregiver preference [13];
- Equity stratification where appropriate (e.g., rurality, Indigenous identity, where ethically and appropriately collected, socioeconomic indicators).

### 8.4. Research Guardrails: Designing Studies That Can Support the Claims Sought

To support implementation claims, future pragmatic trials and observational studies should more consistently include:

- Comparator arms reflecting real choices (e.g., stepped-care, restorative alternatives where feasible) [10,16];
- Calibrated outcome assessment and analytic plans addressing clustering [5,6,7].
- Longer horizons with clinically meaningful outcomes (reactivation, tooth survival, OR rehabilitation rates);
- Lesion-site sampling and pre-specified analytic pipelines when mechanistic claims are advanced, consistent with microbiome reporting standards [19,20].

Future pragmatic trials and implementation studies should include longer follow-up horizons, clinically meaningful outcomes, and analytic strategies that address clustering and outcome assessment bias [5,6,7,19,20].

The practical guardrails proposed to prevent evidence-to-implementation drift in SDF-first and delegated ECC pathways are summarized in Table 1 [33,34].

## 9. Conclusions

SDF is a valuable non-restorative option for arresting caries and supporting access, and screening/risk tools can strengthen early identification when integrated into coherent systems. The key issue is translation: short-horizon arrest outcomes, exploratory mechanistic observations, and screening tool validation should not be over-extended into claims that SDF-first delegated pathways can substitute for dentist-led diagnosis, pulpal assessment, and definitive rehabilitation.

A responsible implementation approach is feasible: position SDF as a bridge within a continuum of care, build time-bound referral pathways with completion tracking, define follow-up and escalation protocols, and evaluate programs using outcomes that reflect children’s lived experience, long-term tooth health, and equity. Guardrails do not slow progress; they ensure that access innovations expand care without lowering the ceiling for children who already bear the greatest burden.

## Figures and Tables

**Table 1:** Practical guardrails to prevent evidence-to-implementation drift in SDF-first and delegated ECC pathways.

SDF use	Arrest treated as “treatment completion”	SDF positioned as interim management within a pathway	Reassess response within 4–8 weeks in high-risk children; reapply/monitor at 3–6 months based on risk/lesion activity
Diagnosis and planning	Screening/checklists treated as diagnosis	Dentist-led accountability for treatment planning when cavitated lesions/pulpal risk are possible	Explicit escalation criteria; defined pathways for dentist review
Follow-up	Single-visit models with loss to follow-up	Protocolized recall aligned to risk	Follow-up intervals defined by risk and lesion status; missed follow-ups trigger outreach
Referral systems	“Referral written” counted as success	Completion tracking and time-bound referrals	Measure completed dental assessment/treatment; audit and report completion rates
Communication	Preventive actions interpreted as “dental care completed”	Standardized caregiver messaging	Scripts/handouts clarifying what SDF/screening does and does not replace
Outcomes	Surrogate endpoints dominate evaluation	Core outcome set beyond arrest	Pain/function, OHRQoL domains, unplanned visits, tooth survival, time-to-definitive care, equity stratification
Mechanistic claims	Non-site-specific sampling used to justify clinical simplification	Sampling and inference alignment	Lesion-site sampling if lesion-level inference is claimed; preregister pipelines
Equity	Reduced standard normalized for high-burden groups	Equity as access to full spectrum of care	Track dental home establishment, treatment completion, OR rates, and outcomes across groups

## Abbreviations

AAPD	American Academy of Pediatric Dentistry
ADA	American Dental Association
ART	Atraumatic Restorative Treatment
CONSORT	Consolidated Standards of Reporting Trials
CRA	Caries Risk Assessment
CCRAT	Canadian Caries Risk Assessment Tool
ECC	Early Childhood Caries
HPCDP	Health Promotion and Chronic Disease Prevention in Canada
NaF	Sodium Fluoride
OHRQoL	Oral Health-Related Quality of Life
ROB-2 (RoB 2)	Revised Cochrane Risk of Bias Tool for Randomized Trials
SDF	Silver Diamine Fluoride
STROBE	Strengthening the Reporting of Observational Studies in Epidemiology
